# The Structure and Biological Function of CREG

**DOI:** 10.3389/fcell.2018.00136

**Published:** 2018-10-26

**Authors:** Gaby Ghobrial, Luiz Araujo, Felecia Jinwala, Shaohua Li, Leonard Y. Lee

**Affiliations:** Department of Surgery, Robert Wood Johnson Medical School, Rutgers, The State University of New Jersey, New Brunswick, NJ, United States

**Keywords:** CREG, mannose-6-phosphate/insulin-like growth factor-2 receptor, exocyst, lysosome, proliferation, differentiation, metabolism

## Abstract

The cellular repressor of E1A-stimulated genes (CREG) is a 220 amino acid glycoprotein structurally similar to oxidoreductases. However, CREG does not have enzymatic activities because it cannot bind to the cofactor flavin mononucleotide. Although CREG can be secreted, it is mainly an intracellular protein localized in the endocytic-lysosomal compartment. It undergoes proteolytic maturation mediated by lysosomal cysteine proteases. Biochemical studies have demonstrated that CREG interacts with mannose-6-phosphate/insulin-like growth factor-2 receptor (M6P/IGF2R) and exocyst Sec8. CREG inhibits proliferation and induces differentiation and senescence when overexpressed in cultured cells. In Drosophila, RNAi-mediated knockdown of CREG causes developmental lethality at the pupal stage. In mice, global deletion of the CREG1 gene leads to early embryonic death. These findings establish an essential role for CREG in development. CREG1 haploinsufficient and liver-specific knockout mice are susceptible to high fat diet-induced obesity, hepatic steatosis and insulin resistance. The purpose of this review is to provide an overview of what we know about the biochemistry and biology of CREG and to discuss the important questions that remain to be addressed in the future.

## Introduction

The adenovirus E1A protein promotes cellular proliferation and inhibits differentiation through its interaction with many cellular proteins including retinoblastoma (pRb) (Chakraborty and Tansey, 2009). Veal et al. (1998) identified human cellular repressor of E1A-stimulated genes (CREG) in yeast two-hybrid screening of a Drosophila cDNA library. It was first described as a transcription repressor that represses the transcription and cellular transformation induced by the E1A oncoprotein. Later CREG was found to be a glycoprotein and could be secreted, when immunoprecipitation experiments confirmed the presence of CREG in conditioned media from transfected cells (Veal et al., 2000). CREG has a signal peptide (amino acids 1–31 in human and mice, 1–23 amino acids in Drosophila) and one or more N-glycosylation sites (three in human, N160, N193, and N216; two in mice, N160 and N216; and one in Drosophila, N87) (Veal et al., 1998; Kowalewski-Nimmerfall et al., 2014). It is modified by cleavage and N-glycosylation after translation. When overexpressed or added to the cells as a conditioned medium, CREG was shown to augment the differentiation of embryonal carcinoma cells (Veal et al., 2000). Most recently, CREG has been identified as a residential protein of the endocytic-lysosomal compartment (Schahs et al., 2008; Kowalewski-Nimmerfall et al., 2014). To date, much progress has been made in our understanding of the structure, biochemical modifications, subcellular localization, and biological activities of this mysterious protein. Genetic analysis revealed a critical role of CREG in Drosophila and mammalian development. However, the underlying cellular mechanisms and molecular mechanisms remain to be explored.

## The Structure and Biochemistry of CREG

Cellular repressor of E1A-stimulated genes is conserved throughout evolution. Drosophila has a single CREG gene while human and mice also express CREG2, which shares a 35% identity with the CREG1 protein (Kunita et al., 2002; Kowalewski-Nimmerfall et al., 2014). In mice, CREG1 is expressed in many tissues including the spleen, liver, kidneys, lungs, heart, and fat tissue (Veal et al., 2000). Its expression is upregulated during embryonic development. CREG2 is only expressed in the brain (Kunita et al., 2002). These results suggest that CREG may play a vital role in development and normal physiology.

In 2005, the crystal structure of CREG1 was solved by Sacher et al. (2005). It is a pyrid oxidase 2 domain-containing protein. However, unlike pyridoxine 5′-phosphate oxidase the flavin mononucleotide (FMN) binding cleft in CREG1 is blocked and CREG1 does not bind to FMN *in vitro*, suggesting that CREG1 is unlikely to have oxidoreductase activities. Similar to pyridoxine 5′-phosphate oxidase, CREG1 was found to associate into tightly bound dimers, with each monomer forming a split β-barrel structure. The surface of the monomer itself is varied; one side is smooth and concave, and serves as the point of dimerization, while the other areas of the protein are ragged and may mediate protein-protein interactions.

The dimer surface is mostly hydrophobic, similar to the monomer interior. Nearly twenty percent of the surface area of the CREG1 molecule is involved with dimerization (Sacher et al., 2005). All three glycosylation sites of human CREG are exposed on the dimer surface. As CREG1 binds to cation-independent mannose-6-phosphate/insulin like growth factor 2 receptor (M6P/IGF2R) (as described below), dimerization may increase the affinity of this interaction and promote the formation of the ligand-receptor clusters.

Cellular repressor of E1A-stimulated genes is modified by N-linked glycosylation in the endoplasmic reticulum (ER) after translation. Treatment of cell lysates with peptide-N-glycosidase F that cleaves off the N-glycans significantly reduced the apparent molecular mass of CREG (Veal et al., 2000; Schahs et al., 2008). *In vitro* translation experiments in the presence of microsomes showed that deletion of the N-terminal signal peptide blocked CREG glycosylation. In addition, the signal peptide is required for CREG secretion into the medium (Veal et al., 2000). This suggests that the signal sequence ensures CREG to enter the ER lumen. Mass spectrometric analysis confirmed that both human and mouse CREG contain phosphorylated N-glycans including the mannose-6-phosphate residue (Schahs et al., 2008). The latter is a high affinity ligand for both M6P/IGF2R and cation-dependent mannose-6-phosphate (M6P) receptor. In addition to the glycosylation modification, intracellular CREG is further proteolytically processed at the N-terminus into smaller molecular sizes by lysosomal cysteine proteases. The biological significance of this modification is unknown.

## CREG Subcellular Localization

Computational sequence prediction indicates a signal peptide at the N-terminus of human and mouse CREG1. The signal peptide is expected to be cleaved between amino acid position 31 and 32 (SignalP4.1). Therefore, CREG is predicted be a secreted protein. Indeed, we and others detected CREG1 in conditioned media of cells overexpressing CREG1 (Veal et al., 2000). One study estimated only 5% of total CREG1 detected in NIH3T3 fibroblast conditioned media (Schahs et al., 2008). The majority of CREG1 was intracellular. CREG1 intracellular retention depends on M6P receptors, which transport lysosomal enzymes from Golgi to lysosomes (Kollmann et al., 2005; Qian et al., 2008; Schahs et al., 2008). Knockout of M6P receptors in mice led to increased blood CREG levels (Qian et al., 2008). Fibroblasts that lack both M6P receptors released most of CREG into the culture medium (Schahs et al., 2008). Proteomics analysis of M6P receptor affinity purified proteins revealed coexisting of CREG with lysosomal proteins (Journet et al., 2000, 2002; Kollmann et al., 2005; Qian et al., 2008). These findings suggest that CREG is a candidate lysosomal protein. CREG localization has also been studied by immunofluorescence microscopy. Earlier studies showed that overexpressed CREG was localized in a perinuclear pattern, similar to that of ER markers (Veal et al., 2000). Schahs et al. (2008) expressed a CREG-GFP fusion protein in fibroblasts and showed co-localization of CREG-GFP with lysosomal cathepsin D. The authors were also able to detect co-localization of CREG with the lysosomal membrane marker LAMP-1 using self-generated anti-CREG antibody in non-transfected fibroblasts. The immunofluorescence findings are supported by biochemical fractionation that showed co-sedimentation of CREG with lysosomal proteins. In addition, treatment of cells with NH_4_Cl, a lysosomotropic agent, increased CREG release into the culture medium. All the above evidence suggests that CREG is a residential protein of the endocytic-lysosomal system. Drosophila CREG, which carries one N-glycosylation site, has also been shown to reside in the lysosome (Kowalewski-Nimmerfall et al., 2014). However, mutation of the glycosylation site or knockdown of lysosomal enzyme receptor protein (closely related to mammalian M6P/IGF2R but unable to bind M6P) does not affect CREG targeting to the lysosome. This demonstrates that CREG can be delivered to the lysosome in an M6P-independent fashion.

## The Biological Activity of CREG

### The Role of CREG in Cell Growth and Proliferation

Cellular repressor of E1A-stimulated genes was first described as an antagonist to adenovirus E1A and H-Ras-induced transformation of primary baby rat kidney (BRK) cells (Veal et al., 1998). When co-transfected with these oncogenes, CREG inhibited BRK cell proliferation into foci that lost contact inhibition. In human teratocarcinoma NTERA-2 cells, overexpression of CREG decreased cell proliferation by ∼60% with significant increase of cells in the G1 phase and decrease of cells in the S/G2 phase compared to the control (Di Bacco and Gill, 2003). By contrast, CREG knockdown in NIH3T3 fibroblasts had a significant increase in cell number in the S and G2/M phase, suggesting enhanced proliferation (Han Y. L. et al., 2008). Moolmuang and Tainsky (2011) found that CREG expression was decreased during immortalization of Li-Fraumeni Syndrome (LFS) fibroblasts. Forced expression of CREG in the immortal LFS cells suppressed cell proliferation. In line with these findings, CREG was shown to inhibit proliferation of human artery smooth muscle cells *in vitro* (Han Y. et al., 2008). In rat neonatal cardiomyocytes, CREG overexpression suppressed cell growth (Xu et al., 2004). In mouse model of cardiac hypertrophy caused by pressure overloading, transgenic expression of CREG attenuated cardiac hypertrophy (Bian et al., 2009). This was also accompanied by a significant reduction in the severity of cardiac fibrosis. Although these studies indicate that CREG is a negative regulator of cell growth and proliferation, the underlying molecular mechanisms are largely unknown. It has been suggested that the CREG interaction with M6P/IGF2R is required for its growth inhibitory effect (Di Bacco and Gill, 2003). The signaling molecules more distal in various pathways, such as MAP kinases might also be involved (Xu et al., 2004).

In contrast to the aforementioned studies, CREG has been suggested to mediate glucocorticoid-induced rat neonatal ileal mucosa hypertrophy (Gordon et al., 2005). In ileal epithelial cells, CREG expression was induced by dexamethasone in parallel with increased IGF2R degradation. Treatment of the cells with CREG antibody blocked dexamethasone-induced IGF2R degradation. The authors suggest that CREG promotes ileal epithelial cell proliferation by inducing the degradation of the IGF-2 scavenger receptor IGF2R, thereby increasing the extracellular IGF2 concentration. In gastric cancer patients, the expression of CREG was higher in tumors than adjacent normal tissues (Xu et al., 2011). Similarly, CREG levels were elevated in human gastric cancer cell lines compared with normal gastric cells. siRNA-mediated knockdown of CREG inhibited cancer cell proliferation. In a SILAC (stable isotope labeling with amino acids in cell culture) proteomic study of non-small cell lung cancer cell lines, Clark et al. (2016) found that CREG was highly expressed and closely correlated with K-Ras mutations in lung cancer cells. siRNA knockdown of K-Ras suppressed CREG expression, while depletion of CREG in the K-Ras mutant lung cancer cell lines inhibited proliferation. Why CREG exerts an opposite effect on cell growth in ileal epithelial cells and cancer cells remains to be elucidated. It is important to determine the role of CREG in growth and proliferation *in vivo* using knockout and transgenic mice.

### The Role of CREG in Cellular Differentiation and Senescence

Early studies showed that the expression level of CREG was low in undifferentiated embryonic stem cells and embryonal carcinoma cells and was rapidly upregulated upon cellular differentiation (Veal et al., 2000). Overexpression of CREG augmented retinoic acid-induced differentiation of embryonal carcinoma cells into the neuronal lineage while suppressed the expression of the pluripotency marker SSEA3. Similarly, CREG expression was induced during the differentiation of human vascular smooth muscle cells (Han et al., 2006). Overexpression of CREG promoted serum starvation-induced smooth muscle differentiation, whereas shRNA-mediated CREG knockdown inhibited differentiation (Han Y. et al., 2008). By using gain- and loss-of-function analysis, we have also shown that CREG induced cardiomyogenic differentiation from embryonic stem cells (Liu et al., 2016). The study on embryonal carcinoma cells suggests that CREG could induce differentiation as a soluble factor (Veal et al., 2000). Whether this differentiation effect is mediated by the CREG interaction with IGF2R remains to be determined.

Fibroblasts derived from patients with LFS, an inherited cancer syndrome linked to germline mutations of the tumor suppressor gene *TP53*, spontaneously undergo immortalization accompanied by CREG downregulation, which could be reversed by treatment with the DNA methyltransferase inhibitor 5-aza-deoxycytidine (Moolmuang and Tainsky, 2011). This suggests that CREG expression is epigenetically silenced by DNA methylation. 5-aza-deoxycytidine also induced cellular senescence of the immortalized fibroblasts. Ectopic expression of CREG together with the tumor suppressor p16INK4a reduced cell proliferation and induced cell cycle arrest accompanied by cellular senescence. It remains to be elucidated how CREG enhances p16INK4a-induced senescence.

### The *in vivo* Function of CREG

Drosophila has only one CREG orthologous gene which encodes a protein containing 211 amino acid residues with 55% similar to human CREG1 (Veal et al., 1998). Kowalewski-Nimmerfall et al. (2014) depleted CREG in Drosophila using the RNAi technology and found that Drosophila development arrested at the pupal stage when CREG protein expression was reduced by >95%. Introduction of a CREG-GFP fusion protein partially rescued the lethal phenotype. This result suggests that CREG is required for Drosophila development to the adult stage. Given that Drosophila CREG also resides in the lysosome, the author further studied its function in lysosomes and autophagy in fat bodies, which store and release energy in response to energy demands. LysoTracker staining of the lysosome was significantly reduced in CREG knockdown larval fat bodies in response to starvation. However, the expression of ATG8-GFP was unchanged, indicating that the formation of autophagosomes is not affected by CREG depletion. It would be interesting to further explore the role of CREG in lysosomal biogenesis/function and autophagy flux downstream of autophagosome formation.

In an attempt to elucidate the function of CREG in the heart, Bian et al. (2009) overexpressed human CREG in mice under the control of α-myosin heavy chain promoter,. Twofold increase in CREG protein expression in the heart had no adverse impact on development, reproduction and longevity as well as heart histology and function. However, it significantly inhibited aortic banding and angiotensin II induced cardiac hypertrophy and fibrosis. This effect was attributed to a complete inhibition of MEK-ERK1/2 MAP kinase and SMAD-2 activation. In addition, angiotensin and pressure overload induced production of inflammatory cytokines including tumor necrosis factor-α, interleukin-1 and interleukin-5 was markedly attenuated by CREG overexpression. How increase of CREG alters multiple signaling pathways in cardiac pathology remains to be addressed. The role of CREG in angiotensin-induced cardiac fibrosis was also studied in CREG heterozygous mice (CREG1 homozygous null mice die during embryonic development) (Yan et al., 2015). In these mice, CREG1 expression was reduced and cardiac fibrosis was augmented compared with wild-type littermates. The increased cardiac fibrosis was associated with decreased autophagy and Rab7 expression (Yan et al., 2015). The authors proposed that impaired autophagy in the CREG heterozygous heart enhanced angiotensin-induced cardiac remodeling. However, pressure overload induced similar cardiac remodeling in cardiac-specific ATG5 knockout and control mice (Nakai et al., 2007). This suggests that defective autophagy may not be the main cause of increased cardiac fibrosis in CREG heterozygous mice. CREG was also shown to suppress angiotensin II-induced remodeling of the arterial wall in mice and rats (Li et al., 2017). This effect may be mediated by soluble CREG since delivery of recombinant CREG to CREG heterozygous mice via an osmotic pump attenuated vascular remodeling.

Cellular repressor of E1A-stimulated genes 1 is ubiquitously expressed in mouse and human including fat and liver (Veal et al., 2000; Tian et al., 2017). Tian et al. (2017) observed that CREG heterozygous mice fed with high fat diet displayed a prominent obese phenotype and gained 30% more body weight compared with wild-type controls. The body weight gain was mainly due to increased fat deposition into white adipose tissues. Since food intake by the mutant mice was comparable to the wild-type, the increased fat deposition is likely due to reduced energy expenditure and increased lipid synthesis. A further study using metabolic cages should provide a definitive answer. The mutant mice developed insulin resistance and adipose tissue inflammation. The same group also reported that conditional ablation of CREG in mouse liver caused hepatic steatosis, obesity and insulin resistance when the animals were fed with high fat diet (Zhang et al., 2017). The underlying molecular mechanisms of this metabolic phenotype are unknown. Given that mice with deletion of autophagy and lysosome-related genes are resistant to high fat diet-induced obesity and hepatic steatosis, the CREG effect is unlikely to be mediated by reduced autophagy and/or lysosomal function (Zhang et al., 2009; Yasuda-Yamahara et al., 2015).

Recently mouse embryonic stem (ES) cells overexpressing CREG were shown to reduce infarction area and improve cardiac function after transplanted into infarcted mouse hearts (Zhang et al., 2018). A significant finding of this study is that CREG overexpression suppressed teratoma formation of ES cells in the infarcted heart. In addition, these genetically modified cells inhibited inflammatory responses and cardiomyocyte apoptosis. It is not addressed whether CREG-expressing cells improve myocyte survival through cytokine reduction and whether decreased myocyte apoptosis contributes to the improvement of cardiac function. The authors also found that CREG overexpression in ES cells increased cardiomyocyte differentiation in embryoid body cultures. Given that overexpression of CREG inhibited teratoma formation, it warrants further investigation into the fate of transplanted CREG ES cells to determine whether they are committed to the cardiac lineage in the cardiac tissue microenvironment.

## CREG Interactions With M6P/IGF2R and the Exocyst

The structural characteristics of the CREG1 molecule have implications for its receptor binding activity. Specifically, human CREG1 has three *N*-glycosylation sites exposed on the surface of the CREG1 dimer. These glycosylation sites may provide an interface for which the CREG1 molecule can interact with M6P/IGF2R. Co-immunoprecipitation and overlay studies have shown that CREG binds its putative receptor M6P/IGF2R (Di Bacco and Gill, 2003). CREG has been described to bind M6P/IGF2R extracellular domains 7–10 in a glycosylation-dependent manner, while binding domains 11–13 in a glycosylation-independent manner (Abdul-Ghani et al., 2011; Han et al., 2011). However, the binding experiment was performed using synthetic M6P/IGF2R peptide fragments on the solid phase. The exposure of hydrophobic residues buried inside the naturally folded protein may lead to false results. Overlay experiments showed that the extracellular portion of M6P/IGF2R binds glycosylated forms of CREG but not deglycosylated forms and preferentially binds multiglycosylated CREG over monoglycosylated CREG (Di Bacco and Gill, 2003). It is thought that dimerization may increase the affinity of the CREG-IGF2R complex through interactions with distinct mannose 6-phosphate binding sites (Journet et al., 2000; Sacher et al., 2005).

M6P/IGF2R is a scavenger receptor for IGF-2. It is also involved in the transport of acid hydrolases from the trans-Golgi network to lysosomes (Kornfeld, 1992; Hille-Rehfeld, 1995; Le Borgne and Hoflack, 1998; Ghosh et al., 2003). Despite the will-studied function of M6P/IGF2R, little is unknown about the biological effect of the CREG interaction with IGF2R. One study showed that ectopic expression of CREG inhibited the growth of M6P/IGF2R-positive MS9-II cells but had no effect on MS9 cell deficient in M6P/IGF2R (Di Bacco and Gill, 2003). This result suggests that M6P/IGF2R is required for CREG-mediated growth inhibition. It remains to be determined whether CREG exerts its growth suppressive effect through binding to M6P/IGF2R. If so, it would be interesting to know if CREG promotes M6P/IGF2R-dependent IGF-2 internalization and degradation. The CREG-M6P/IGF2R interaction plays an important role in the cellular retention of CREG. In this case M6P/IGF2R and cation-dependent M6P receptor compensate for each other to mediate trafficking of CREG from Golgi to lysosomes (Schahs et al., 2008).

Recently in an unbiased affinity pull-down assay followed by mass spectrometry, we identified Sec8 (exocyst complex component 4, Exoc4) as a binding partner of CREG1 in addition to M6P/IGF2R in the embryonic heart (Liu et al., 2016). The exocyst is an octameric protein complex composed of Sec3 (Exoc1), Sec5 (Exoc2), Sec6 (Exoc3), Sec8, Sec10 (Exoc5), Sec15 (Exoc6), Exo70 (Exoc7), and Exo84 (Exoc8). It is required for the tethering of vesicles to the plasma membrane prior to vesicle fusion (Wu and Guo, 2015). The exocyst is enriched in the region of membrane growth and at cell-cell junctions. It also has a role within the endocytic system (Grote et al., 2000; Langevin et al., 2005; Oztan et al., 2007). We showed that CREG1 binds to Sec8 and co-localized with N-cadherin at an intracellular vesicular compartment and intercalated disks in cardiomyocytes. We further showed that CREG1 was upregulated during the differentiation of embryonic stem (ES) cells into cardiomyocytes and its expression level correlated with that of cardiomyogenic markers. Overexpression of CREG1 in ES cells promoted cardiomyocyte differentiation and the formation of a cohesive myocardium-like structure, whereas CREG knockout had inhibitory effects. Site-directed mutagenesis and rescue experiments demonstrated that CREG1 binding to Sec8 is required for cardiomyocyte differentiation from ES cells and the formation of a cohesive myocardium-like structure. These findings suggest the CREG1 may be involved in the formation of intercalated disks between cardiomyocytes, which are vital to the synchronized contraction of cardiomyocytes. Previously, stem cell-derived cardiomyocytes would often fail to form cell-cell junctions after being engrafted in normal cardiac muscle (Passier et al., 2008). If CREG1 promotes intercalated disk assembly, it may be a target for improving the transplantation of stem cell-derived cardiomyocytes into recipient hearts. At present, we are in the process of determine the role of CREG1 in cardiac development and maintenance using conditional knockout mice.

## Concluding Remarks and Future Directions

**FIGURE 1:**
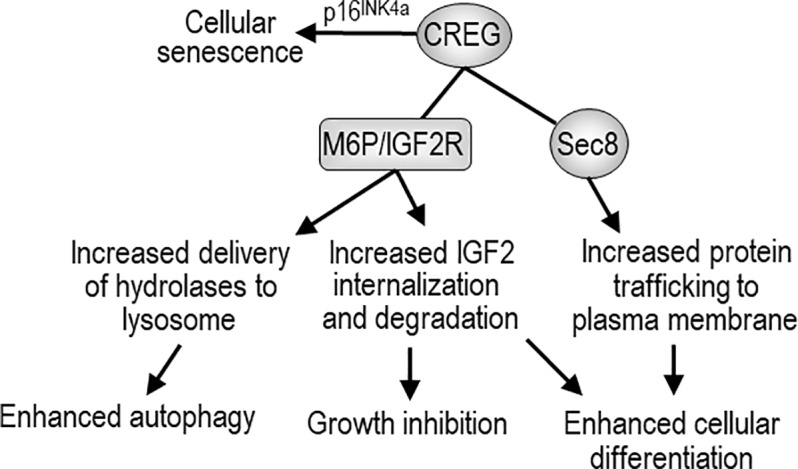
The biological activity of CREG and possible mechanisms of action. CREG binds to mannose-6-phosphate/insulin-like growth factor-2 receptor (M6P/IGF2R) and may enhance autophagy through M6P/IGF2R-mediated delivery of lysosomal hydrolases from Golgi to the lysosome. CREG may also facilitate M6P/IGF2R-mediated internalization and degradation of extracellular IGF-2, thereby inhibiting cell growth and promoting differentiation. CREG interacts with the exocyst component Sec8 and promotes cellular differentiation and cell-cell junction formation. In addition, CREG acts synergistically with p16^INK4a^ to induce cellular senescence.

Cellular repressor of E1A-stimulated genes is a M6P-containing glycoprotein essential for Drosophila and mouse development. Since it was cloned in 1998, Progress has been made in our understanding of its structure, biochemical properties, biological activities, and even involvement in pathological processes (Figure 1). However, the mechanisms whereby CREG regulates embryonic development and the reported cellular activities are still unknown. In this regard, detailed analysis of the lethal phenotype should shed light on how CREG works at the cellular and molecular level. Cell culture studies demonstrated a role for CREG1 in growth, differentiation and senescence. These biological activities need to be confirmed *in vivo* by gene targeting and transgenic expression. A pressing immediate question is how CREG as an endosomal/lysosomal protein regulates these cellular activities. The endosomal-lysosomal system has been shown to play an important signaling role in the activation and degradation of signaling molecules (Dobrowolski and De Robertis, 2011; Sun-Wada and Wada, 2015; Bergeron et al., 2016; Bakker et al., 2017). It also controls the activation of the mechanistic target of rapamycin complex 1 (mTORC1) (Huber and Teis, 2016; Lim and Zoncu, 2016; Perera and Zoncu, 2016). Whether CREG regulates these processes warrant future investigation. In addition, it would be interesting to know if CREG binding to M6P/IGF2R influences IGF2R-mediated internalization of IGF2 and lysosomal enzyme trafficking from Golgi to lysosomes. Structural and biochemical analysis revealed that CREG forms a homodimer. What is the function of dimerization? Because of the lack of fundamental mechanistic studies, it is difficult to provide a unified explanation of its diverse biological activities. With CREG conditional knockout and transgenic mice generated, we begin to address its physiological functions and the underlying mechanisms. These models also allow us to study the possible role of CREG in pathological processes such as infection and cancer.

## Author Contributions

GG, LA, FJ, and SL reviewed the literature and wrote the manuscript. LL wrote the manuscript.

## Conflict of Interest Statement

The authors declare that the research was conducted in the absence of any commercial or financial relationships that could be construed as a potential conflict of interest.
